# Sleep Quality and Disturbances in Tunisia: Insights from the Pittsburgh Sleep Quality Index (PSQI)

**DOI:** 10.1192/j.eurpsy.2026.12097

**Published:** 2026-07-31

**Authors:** K. Mahfoudh, S. Hamzaoui, A. Jaoua, M. Mrad, R. Hosni, A. Ouertani, A. Aissa, R. Jomli

**Affiliations:** Avicenne, Razi Hospital, Manouba, Tunisia

## Abstract

**Introduction:**

Sleep disorders are increasingly prevalent and often underestimated in the general population. Their consequences include cognitive impairment, mood disturbances, and an increased tendency to self-medicate, particularly in contexts where access to specialized care is limited.

**Objectives:**

This study aims to assess sleep quality and characterize sleep disturbances among a Tunisian population using the Pittsburgh Sleep Quality Index (PSQI).

**Methods:**

A cross-sectional descriptive study was conducted between august and november 2024 involving a sample of 287 participants from the general Tunisian population. Data were collected via an online questionnaire, including sociodemographic variables and the validated Arabic version of the PSQI. A PSQI global score ≥6 was considered indicative of poor sleep quality.

**Results:**

The study included a total of 287 participants from the general Tunisian population. The majority of participants (52.96%) were aged between 26 and 35 years, with a gender distribution of 60.98% female. In terms of marital status, 41.81% of participants were single and 39.02% were married. Most participants (88.15%) had a university-level education. Regarding employment status, 65.16% were employed.

The majority of participants reported experiencing sleep disturbances, with the global PSQI score averaging 11.55 ± 3.5. A total of 33.79% of participants scored ≥6 on the PSQI, indicating poor sleep quality.

As for sleep patterns, 49.1% of participants went to bed between 9:00 PM and midnight, while 47.4% went to bed between midnight and 4:00 AM. Regarding wake-up times, 69.7% of participants woke up between 4:00 AM and 8:00 AM, while 24% woke up between 8:00 AM and noon.

Regarding sleep duration, 34.14% of participants reported sleeping between 6 and 7 hours per night, and 30.66% reported sleeping more than 7 hours. However, 13.93% of participants reported sleeping less than 5 hours on average.

Almost half of the participants (55.5%) reported chronic insomnia.

**Conclusions:**

The study highlights the significant prevalence of poor sleep quality and disturbances within the Tunisian population. Addressing these issues could improve public health outcomes, with a focus on enhancing awareness and intervention strategies for better sleep hygiene and management of sleep disorders.

**Disclosure of Interest:**

None Declared

